# Design Constraints on a Synthetic Metabolism

**DOI:** 10.1371/journal.pone.0039903

**Published:** 2012-06-29

**Authors:** Tugce Bilgin, Andreas Wagner

**Affiliations:** 1 Institute of Evolutionary Biology and Environmental Sciences, University of Zurich, Zürich, Switzerland; 2 The Swiss Institute of Bioinformatics, Zurich, Switzerland; 3 The Santa Fe Institute, Santa Fe, New Mexico, United States of America; Umeå University, Sweden

## Abstract

A metabolism is a complex network of chemical reactions that converts sources of energy and chemical elements into biomass and other molecules. To design a metabolism from scratch and to implement it in a synthetic genome is almost within technological reach. Ideally, a synthetic metabolism should be able to synthesize a desired spectrum of molecules at a high rate, from multiple different nutrients, while using few chemical reactions, and producing little or no waste. Not all of these properties are achievable simultaneously. We here use a recently developed technique to create random metabolic networks with pre-specified properties to quantify trade-offs between these and other properties. We find that for every additional molecule to be synthesized a network needs on average three additional reactions. For every additional carbon source to be utilized, it needs on average two additional reactions. Networks able to synthesize 20 biomass molecules from each of 20 alternative sole carbon sources need to have at least 260 reactions. This number increases to 518 reactions for networks that can synthesize more than 60 molecules from each of 80 carbon sources. The maximally achievable rate of biosynthesis decreases by approximately 5 percent for every additional molecule to be synthesized. Biochemically related molecules can be synthesized at higher rates, because their synthesis produces less waste. Overall, the variables we study can explain 87 percent of variation in network size and 84 percent of the variation in synthesis rate. The constraints we identify prescribe broad boundary conditions that can help to guide synthetic metabolism design.

## Introduction

Among the most important goals of synthetic biology and biotechnology is to engineer organisms with novel properties [1], [2]. Most current efforts focus on designing subsystems of organisms, such as regulatory circuits [1], [3] or metabolic pathways [4], [5]. However, recent advances in genomics and genome synthesis have allowed synthetic biology to make great strides towards the ultimate goal of designing new organisms from scratch [6]–[10].

To be able to synthesize new life, one needs to understand life’s minimal needs. Considerable effort has thus focused on understanding and creating minimal organisms [9]–[15]. One line of research studies organisms with very small genomes that comprise only a few hundred genes, such as the gamma proteobacteria [14], *Blochannia floridanus* [16] and *Carsonella ruddii* [17]. Such organisms are typically endosymbionts or endoparasites, and receive substantial resources from their host [15]. Although valuable knowledge has been gained by studying these organisms, this property renders them of limited use in understanding minimal requirements for a *free-living* organism.

A second, complementary line of research starts from a complex genome, successfully eliminates genes from it without affecting viability, and thus creates a genome that is (close to) minimal. This is possible because free-living organisms have many genes that are dispensable in any one environment [9]–[11], [13], [18]. Such systematic gene deletion efforts not only provide insight into minimal genomes, they can also help to eliminate the synthesis of undesired molecules, avoid excessive waste production, and thus increase the efficiency with which an organism synthesizes desired molecules [19]. Based on both computational and experimental approaches of genome reduction, several proposals for the gene complements of minimal organisms have been made. They range in size from 100 genes to more than 300 genes in organisms such as Mycoplasmas [9], [11], [13].

One indispensable feature of any living organisms is its metabolism. A metabolism is a complex network of chemical reactions, catalyzed by enzymes that are encoded in genes. It uses sources of energy and chemical elements – nutrients – to synthesize molecules that an organism needs, including precursors of biomass and various secondary products, such as molecules for defense and communication [14]. The manipulation of metabolism for technological purposes is known as metabolic engineering [19]–[22].

Metabolic engineering has multiple applications. They include the large-scale, fast, and efficient synthesis of pharmaceuticals, chemical reagents, and biofuels [2], [19], [23]–[26]. The latter class of molecules is especially important given their importance in energy security and in the reduction of greenhouse gas emissions [20], [21], [27], [28]. Another application is bioremediation, where microbes with properly engineered metabolic pathways may be able to clean hazardous waste in inaccessible places [28], [29].

Current metabolic engineering approaches typically manipulate one or a few enzyme-coding genes [1]. Because of the highly interconnected nature of metabolism, and because of the complexity of enzyme regulation, such manipulation faces several challenges. The first is to ensure a high level of expression of the genes and the enzymes they encode. A second challenge is to manipulate cells into selectively producing desired molecules at high rates and yield. Cells can be quite recalcitrant to such manipulations [30]. A third challenge is to ensure that a desired product can be produced from one source of chemical elements and energy, but from multiple sources, to ensure efficient production. For example, yeast species are good candidate organisms to synthesize ethanol, with the drawback that they are not highly efficient at fermenting cellulosic biomass. (In addition to glucose, which yeast strains can catabolize normally, cellulosic biomass contains five carbon sugars, such as arabinose and xylose, which yeast strains cannot catabolize.) Metabolic engineering can create yeast strains that ferment not only glucose but also mixtures of other sugars. [31]. A fourth challenge is to overcome the toxicity of some desired products when they accumulate at high concentrations in a cell. This holds especially for biofuels that are produced in large amounts [27]. Finally, metabolic engineering needs to control ratios of metabolites such as ATP/ADP or NAD+/NADH, which can influence product yields and lead to synthesis of undesired byproducts through their global effects on physiology [21].

While contemporary metabolic engineering focuses on altering existing pathways, future engineering will design metabolisms and minimal organisms de novo [1], [21], [32], [33]. Ongoing technological advances in sequencing and de-novo synthesis and declining prices in these technologies [2], [20], [34], [35] suggest that de-novo synthesis of minimal organisms for biomass production will be feasible soon. A small (synthetic) metabolism may also allow better control of metabolic properties than a large metabolism [1], [18].

To be able to design a metabolism, one needs to be able to predict system-wide metabolic properties. In recent years great strides have been made towards such prediction. Especially noteworthy are constraint-based modeling approaches, which can predict the spectrum of biosynthetic properties of a metabolism from knowledge about the reactions that its enzymes catalyze, and from the nutrients available in its environment [36]–[38]. One such approach, flux balance analysis (FBA) [36], [37], [39], [40], uses information about the stoichiometry of reactions in a metabolic network to predict the rate at which a network can synthesize a given spectrum of molecules, which we refer to as the network’s biosynthetic flux. FBA makes two main assumptions. The first is that a metabolism is in a steady state with a constant nutrient supply. The second is that it maximizes some property, such as biosynthetic flux [41]. While FBA faces challenges caused by regulatory constraints [42], [43], it is well suited to answer simple qualitative and quantitative questions about important properties of a metabolic system [44]. An especially important property is the minimal number of reactions *R* needed to synthesize a given number *B* of (biomass) molecules from a given spectrum *N* of nutrients. The ideal network has few reactions and can synthesize many molecules using a broad spectrum of nutrients. However trade-offs between these properties exist, which do not allow all these requirements to be met.

We here take a first step towards a quantitative understanding of these and other trade-offs using constraint-based methods. To this end, we study the properties of not just one metabolic network, but of multiple networks that differ in these properties. Experimental techniques are not yet suitable to do that, but computational approaches are [45]. The approach we use starts with the observation that any one metabolic network exists in a vast space of possible metabolic networks. The approach uses recently developed techniques [46], [47] to create unbiased arbitrary large samples of networks from this space, where each network of the sample has a specific property, such as a given number of reactions, a given number of carbon sources it can use, and a given set of molecules that it can synthesize. The underlying sampling technique, Markov Chain Monte Carlo sampling [48], [49] is a widely used approach with a well-developed statistical theory [50]–[54]. We use it to quantify the trade-offs and thus the design constraints imposed by important metabolic network properties. Specifically, a first part of our analysis focuses on three main properties. The first is nutrient flexibility *N*, that is, the number of different carbon sources a metabolic network can utilize as *sole* carbon sources. The second is a network’s biosynthetic ability *B*, that is, the number of biomass molecules that it can synthesize. The third is the number of reactions *R* in a network. We then extend our analysis to further properties, such as the biosynthetic flux *S*, the rate at which biomass molecules are synthesized, and the amount *W* of waste produced.

## Results

### Each Additional Nutrient Requires on Average Two Additional Reactions

Due to the presence of alternative metabolic routes that connect many pairs of molecules, most metabolic systems are able to tolerate genotypic changes, such as the deletion of enzyme coding genes. For instance, 80% of single gene deletions in budding yeast have no detectable phenotypic effect in standard laboratory environments [55]. More specifically, metabolic systems are to some extent robust against deletions of enzyme coding genes, because the resulting elimination of metabolic reactions from a metabolic network does not necessarily affect cell viability [9], [11], [13], [23]–[25], [56].

**Figure 1:**
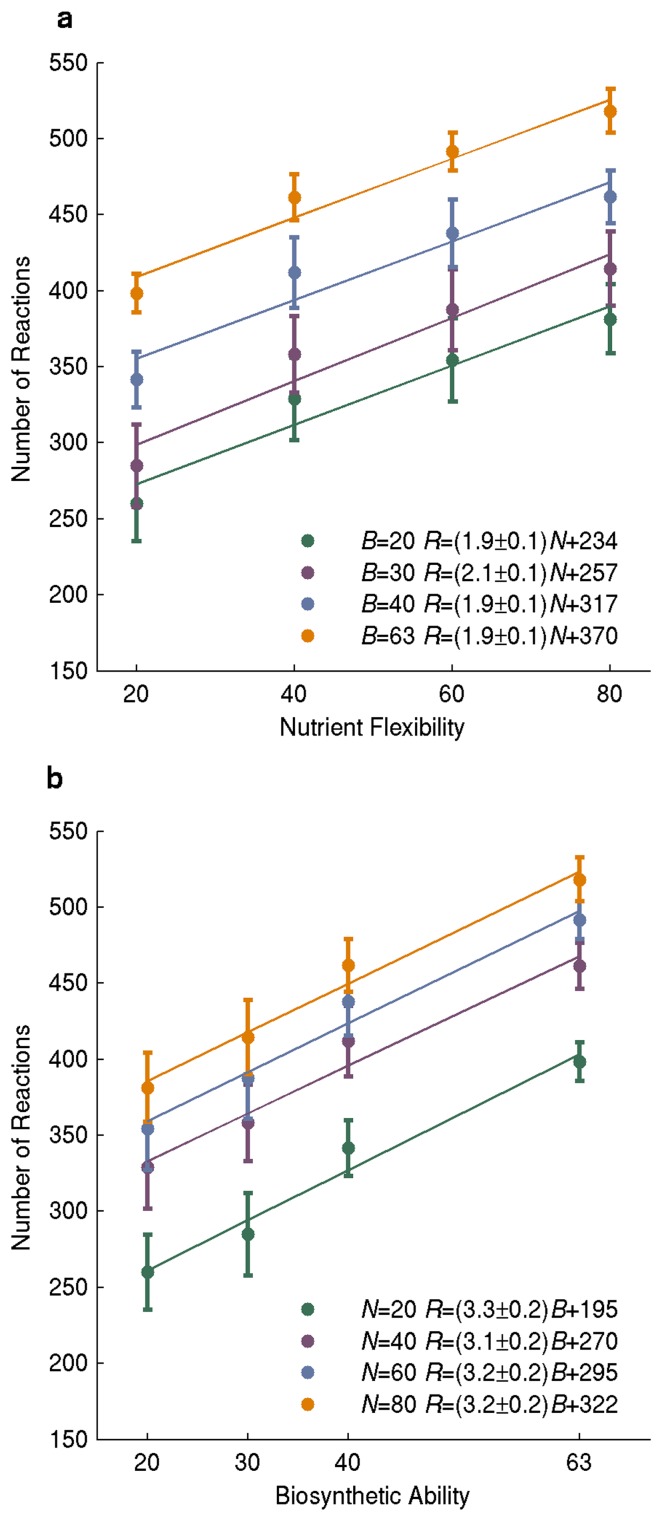
The number of required reactions increases with nutrient flexibility and biosynthetic ability. The vertical axis shows the number of reactions in minimal networks as a function of a) nutrient flexibility and b) biosynthetic ability. Dots and length of error bars correspond to means and one standard deviation based on a sample of n = 50 minimal networks. Solid lines indicate linear regression lines for different values of *B* in a) and *N* in b). Numerical estimates of regression coefficients with 95% confidence intervals are given in the inset, in the format *y = (a±e)x+b*, where *a* is the regression coefficient, *e* the confidence interval, and *b* is the intercept of the regression line with the vertical axis.

We first wanted to explore how the minimally needed number of reactions in a network depends on the network’s nutrient flexibility and its biosynthetic abilities. For example, a network that can synthesize biomass on an increasing number of different carbon sources will require more metabolic reactions. But how many more? To answer this question we created random *minimal* networks (see Methods) with a given nutrient flexibility *N*, that is, networks that can use *N* sole carbon sources to synthesize all biomass components, but in which every single reaction is essential, such that no reaction can be removed without abolishing viability of the network on at least one of the carbon sources. More specifically, we created 50 random viable networks that can use *N* = 20, 40, 60, and 80 carbon sources as sole carbon sources (for a total of 200 networks), and that could synthesize all *B* = 63 *E. coli* biomass components. We then studied the relationship between *N* and the number of reactions in these minimal networks (Figure 1a, orange data points). Linear regression analysis showed that the number of needed reactions increases by a number that is statistically indistinguishable from two for every additional carbon source that a network is required to be viable on. Specifically, we found *R* to depend on *N* as *R* = (1.9±0.1)*N* +234, where the number 0.1 indicates the 95 percent confidence interval of the regression coefficient (see Text S1 for details). We then asked whether the same relationship between *N* and *R* also holds if we vary biosynthetic ability. To this end, we repeated the analysis just described, but for networks that are able to synthesize *B* = 20, B = 30, and B = 40 biomass components. The slope of the regression line was indistinguishable from that of *B* = 63 in all three cases (Figure 1a, green, purple, and blue data; see Figure S3 for distributions of R). In other words, regardless of a network’s biosynthetic abilities, every additional carbon source that a network is required to be viable on requires on average two additional metabolic reactions.

### Each Additional Biomass Molecule Requires Three Additional Reactions on Average

We next analyzed the relationship between biosynthetic ability and the numbers of reactions in greater detail. To this end, we used the same set of random viable minimal networks that we used in the previous analysis. First, we analyzed the number of reactions in 50 random viable minimal networks with the ability to synthesize *B* = 20, 30, 40, or 63 randomly chosen biomass components, that is, we analyzed a total of 200 networks. Each of these networks was required to be viable on *N* = 80 different sole carbon sources. We found that the number of reactions needed for viability under these conditions increased approximately linearly with biosynthetic ability (Figure 1b, orange), such that every additional biomass molecule required approximately 3 additional reactions Specifically, *R* =  (3.3±0.2)*B* +195. We then asked again how this relationship between biosynthetic ability and number of reactions depends on *N*, and thus repeated this analysis for networks viable on *N* = 20, *N* = 40, and *N* = 60 sole carbon sources. The slope of the regression line was indistinguishable for the different values of *N* (Figure 1b, green, purple, and blue data), but the intercept differed, as one might expect from the analysis of the previous section.

The results of pairwise regression analysis are easy to display graphically and to interpret intuitively, which is why we use it here. However, where more than two quantities vary, pairwise regression analysis is insufficient to study dependencies among all of them. We thus also carried out a multiple regression analysis using all 800 minimal networks, where nutrient flexibility and biosynthetic ability were independent variables, and where the reaction number was the dependent variable. Not unexpectedly, the pairwise regression coefficients estimated from the multiple regression analysis were statistically indistinguishable from those estimated from the pairwise analysis above (*R* = 2.0*N*+3.2*B*+171). Overall, variation in the two independent variables explains 87 percent of the variation in the number of reactions of a metabolic network.

Our analysis thus far was only concerned with the qualitative question whether networks can synthesize a given number *B* of molecules, not with the rates at which these molecules can be synthesized. One would therefore expect that our observations are not sensitive to variation in the proportions in which biomass molecules are to be synthesized. An additional analysis (Figure S1) confirms this expectation.

### Network Size does Not Significantly Influence Biosynthetic Flux

Engineered metabolic networks that produce one or more desired products at a high rate are a key goal of biotechnology. For our purpose, it is therefore important to ask whether the maximal rate at which biomass can be synthesized by a network depends on *B* and *N*. We here used the ability of FBA to predict the maximal biosynthetic flux (*S*) of viable networks to answer this question (see Methods).

Our analysis thus far was based on minimal networks, but it is possible that biosynthetic flux depends on the number of reactions in networks that are larger than minimal networks. We therefore first analyzed 1.6×10^4^ random viable networks that are not minimal and that differ in size between *R* = 400 and *R* = 800 reactions. (From here on, we will use the term random networks to refer to those networks that are not minimal, unless stated otherwise.) We found that network size does not influence biosynthetic flux for networks able to synthesize all *B* = 63 *E. coli* biomass molecules. That is, the regression coefficient describing their relationship is statistically indistinguishable from zero (*f* = r*R*, with r = 3×10^−5^±7×10^−5^, *n* = 1000 networks). The same holds for networks that can synthesize *B* = 20, *B* = 30, and *B* = 40 biomass molecules. (See Figure S2 for flux distributions.).

### High Biosynthetic Flux is Usually Achieved by a Modest Number of Active Reactions

We just showed that the number of reactions in a network does not affect its biosynthetic flux. However, it is well-known that only a subset of a network’s metabolic reactions are usually *active* in any one environment, that is, they have nonzero flux [12]. We thus wanted to know whether a relationship exists between biosynthetic flux and the number of active reactions, *R_A_*. This is indeed the case, based on an analysis of all our 1.6×10^4^ random networks viable on glucose as a sole carbon source. More specifically, this relationship is strongly negative (Pearson's r = -0.65 for biosynthetic flux on glucose). That is, the greater the number of active reactions, the lower the biosynthetic flux of a network.

We next used linear regression to ask whether network size *R* itself influences the number of active reactions *R_A_*. The answer is no. Network size has no statistically significant influence on the number of active reactions for any combination of values of *N* and *B*. This observation is in agreement with earlier results by Nishikawa *et al*. [12], which showed that the number of active reactions in a network is not sensitive to the size of the network.

We subsequently asked whether synthesis of each additional biomass molecule also needs more active reactions. The answer is yes, as shown by linear regression analysis (3.6–4 additional reactions, on average, per biomass molecule).

### The More Biomass Molecules a Network Synthesizes, the Smaller is its Biosynthetic Flux

**Figure 2:**
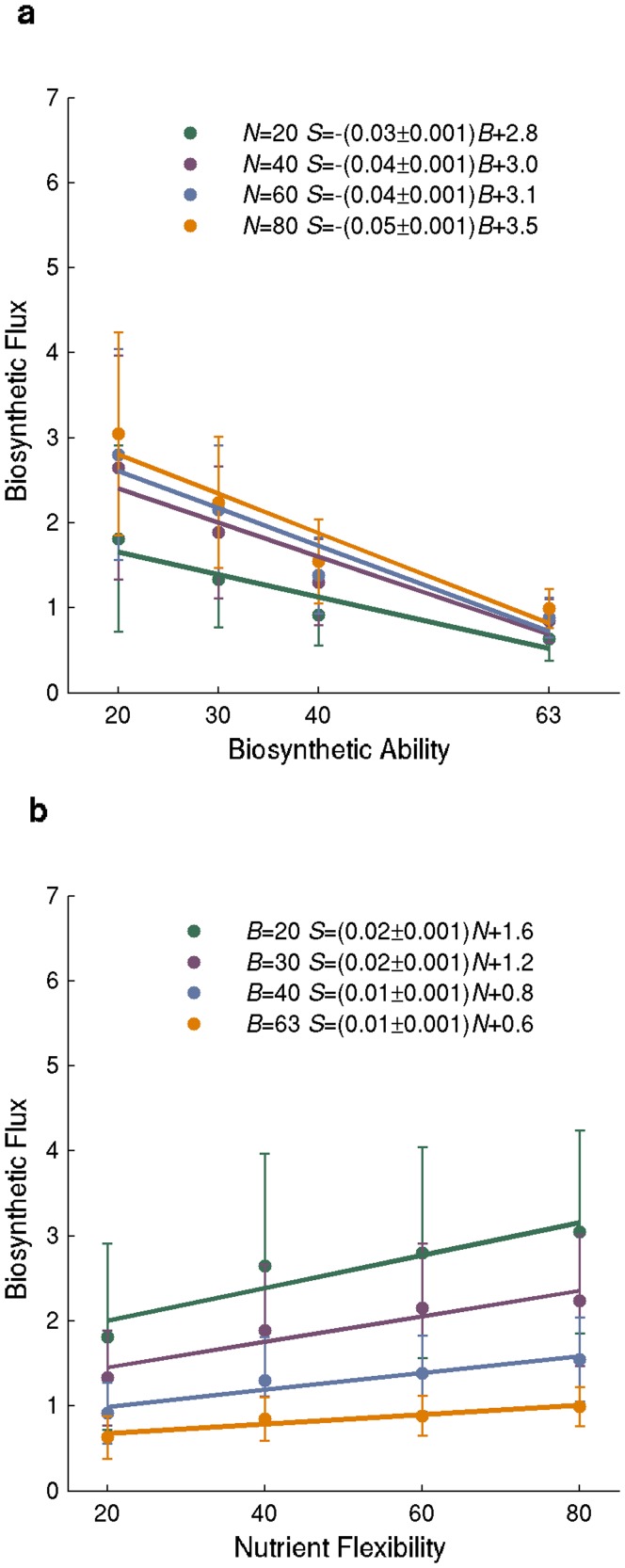
Biosynthetic flux decreases with biosynthetic ability and increases with nutrient flexibility. The vertical axis shows biosynthetic flux in mmoles per g DW per hour in random networks as a function of a) biosynthetic ability and b) nutrient flexibility. Dots and lengths of error bars correspond to means and one standard deviations based on a sample of n = 1000 minimal networks. Solid lines indicate linear regression lines for different values of *B*. Numerical estimates of regression coefficients with 95% confidence intervals are given in the inset, in the format *y = (a±e)x+b*, where *a* is the regression coefficient, *e* the confidence interval, and *b* is the intercept of the regression line with the vertical axis.

We next wanted to explore why the number of active reactions is negatively correlated with biosynthetic flux. Our observations so far show that the number of active reactions increases with biosynthetic ability. We thus hypothesized that increased biosynthetic ability entails smaller biosynthetic flux, because biosynthetic flux should decrease as the number of biomass molecules to be synthesized grows, given a constant nutrient supply. To test this hypothesis, we computed the correlation between the number of molecules synthesized and biosynthetic flux in multiple environments for all 16000 networks we had generated (see Methods). This correlation is significantly negative (Pearson’s r = -0.60; P<10^−300^). Each molecule to be synthesized decreases biosynthetic flux by 0.05 mmoles per g DW per hour, equivalent to a 6 percent decline relative to *E.coli*’s computed biosynthetic flux. Next, we analyzed networks viable on 80 different sole carbon sources that were able to synthesize *B* = 20, 30, 40, or 63 randomly chosen biomass components (1000 networks each, for a total of 4000 networks). Biosynthetic flux under these conditions also decreased approximately linearly with biosynthetic ability (Figure 2a, orange data). Specifically, every additional biomass molecule that a network needs to synthesize decreases the biosynthetic flux by approximately 0.05 mmoles of biomass per g DW per hour (6 percent; *S* = -(0.05±0.001)*B* +3.5). Similar relationships also hold for networks viable on *N* = 20, *N* = 40, and *N* = 60 sole carbon sources (Figure 2a, green, purple, and blue data). They show that, every additional biomass molecule to be synthesized reduces biosynthetic flux by 3–6 percent. Taken together, these observations help explain the negative relation between the number of active reactions and biosynthetic flux. Networks that can synthesize more biomass molecules need more active reactions. Given a constant nutrient supply, the total biosynthetic flux that can be realized by these networks must decrease as the number of molecules that they can synthesize increases. The negative correlation between the number of active reactions and biosynthetic flux is a by-product of the latter two correlations.

### Biosynthetic Flux Increases Weakly with Nutrient Flexibility

We next explored how the nutrient flexibility of a network affects its biosynthetic flux. To this end, we analyzed 1000 networks each that can use *N* = 20, 40, 60, and 80 carbon sources (a total of 4000 networks) and that can synthesize *B* = 63 biomass molecules. (Figure 2b, orange data points). We found that biosynthetic flux increases weakly but significantly with nutrient flexibility (*S* =  (0.01±0.0001)*N* +0.6 mmoles of biomass per g DW hour, n = 1000 networks) for every additional carbon source that a network is viable on. This is equivalent to 1 percent of increase relative to *E. coli*'s biosynthetic flux on glucose. Quantitatively similar relationships hold for networks able to synthesize *B* = 20, *B* = 30, and *B* = 40 biomass components (Figure 2b, green, purple, and blue data). We next turn to a variable that can help explain this association: waste production.

### High Biosynthetic Flux Means Less Waste

Some 300 transport reactions are described in *E. coli* [57], many of which transport waste products either from the periplasm or the cytoplasm to the extracellular space. Metabolic networks may differ in the extent to which they produce waste products that are excreted from cells. Waste products may include molecules that are not biomass molecules (such as carbon dioxide or acetate), as well as excess biomass molecules [12], [57]. Waste production consumes resources, such as organic carbon, and will therefore reduce biosynthetic flux. With these considerations in mind, we next asked whether lower waste production might be responsible for the differences in biosynthetic flux we observed in networks with different nutrient flexibility.

Different metabolic networks may excrete different kinds of molecules, but to compare their waste production, it is useful to establish a common waste ‘currency’. Since our analysis is focused on carbon metabolism, we use the moles of carbon per g DW per hour as our unit of waste production. (Note that these moles of carbon may well come from a broad spectrum of different molecules.).

We first wanted to know if waste has any influence on biosynthetic flux. The answer is yes, based on an analysis of all our 1.6×10^4^ random viable networks on glucose as the sole carbon source. This relationship is strongly negative (Pearson's r = -0.66) That is, the greater waste production is, the lower the biosynthetic flux.

**Figure 3:**
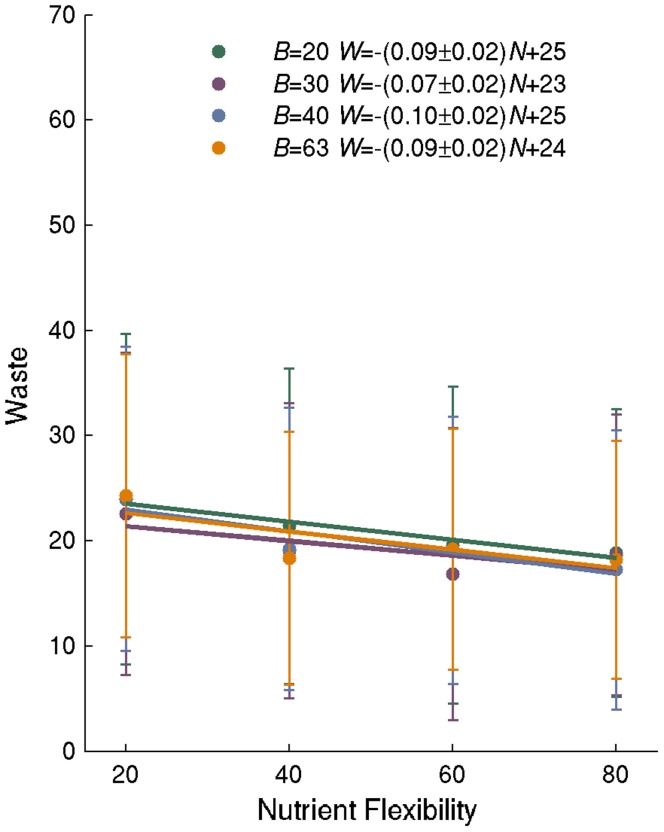
Waste production decreases with nutrient flexibility. The vertical axis shows waste that is excreted carbon in mmoles per g DW hour in random networks as a function of nutrient flexibility. Dots and lengths of error bars correspond to means and one standard deviation based on a sample of n = 1000 minimal networks. Solid lines indicate linear regression lines for different values of *B*. Numerical estimates of regression coefficients with 95% confidence intervals are given in the inset.

In the last section, we showed that for each additional carbon source a network was required to be viable on, biosynthetic flux increased by 0.01–0.02 mmoles of biomass per g DW and hour (Figure 3B). If we express biomass synthesis instead as moles of carbon (in synthesized biomass) per g DW and per hour, biosynthetic flux increases on average by 0.1 mmoles of carbon per g DW hour with increasing nutrient flexibility. We hypothesized that this increase can be explained through the influence of nutrient flexibility *N* on waste production *W*. To this end, we first analyzed random viable networks that vary in *N* and that can synthesize all *B* = 63 molecules *E. coli* biomass molecules (Figure 3, orange data points). We found that waste production *decreased* by a number that is statistically indistinguishable from 0.1 mmol of carbon per g DW hour (*W* = (-0.09±0.02)*N* +24, n = 1000 networks) for every additional carbon source that a network is viable on. Statistically indistinguishable relationships exist for networks with *B* = 20, *B* = 30, and *B* = 40 biomass components. (Figure 3, green, purple, and blue data). These observations suggest that the positive influence of nutrient flexibility on biosynthesis flux comes from reduced waste production. We later discuss experimental evidence supporting this observation.

In a final analysis related to waste production, we studied the relationship between biosynthetic ability and waste production, and found no significant such relationship.

### The Variables We Considered can Explain 84 Percent of the Variance in Biosynthetic Flux

Thus far, we have considered five variables and how they influence biosynthetic flux. These are network size *R*, nutrient flexibility *N*, biosynthetic ability *B*, waste production *W*, and numbers of active reaction *R_A_*. To understand how biosynthetic flux depends on not just one but all of these variables, we carried out a multiple regression analysis with flux as the dependent variable. This regression analysis showed that the variables we analyzed explain 84 percent of the variation in biosynthetic flux (R^2^ = 0.84).

### Synthesis of Biochemically Related Molecules Requires Fewer Reactions, because it Produces Less Waste

**Figure 4:**
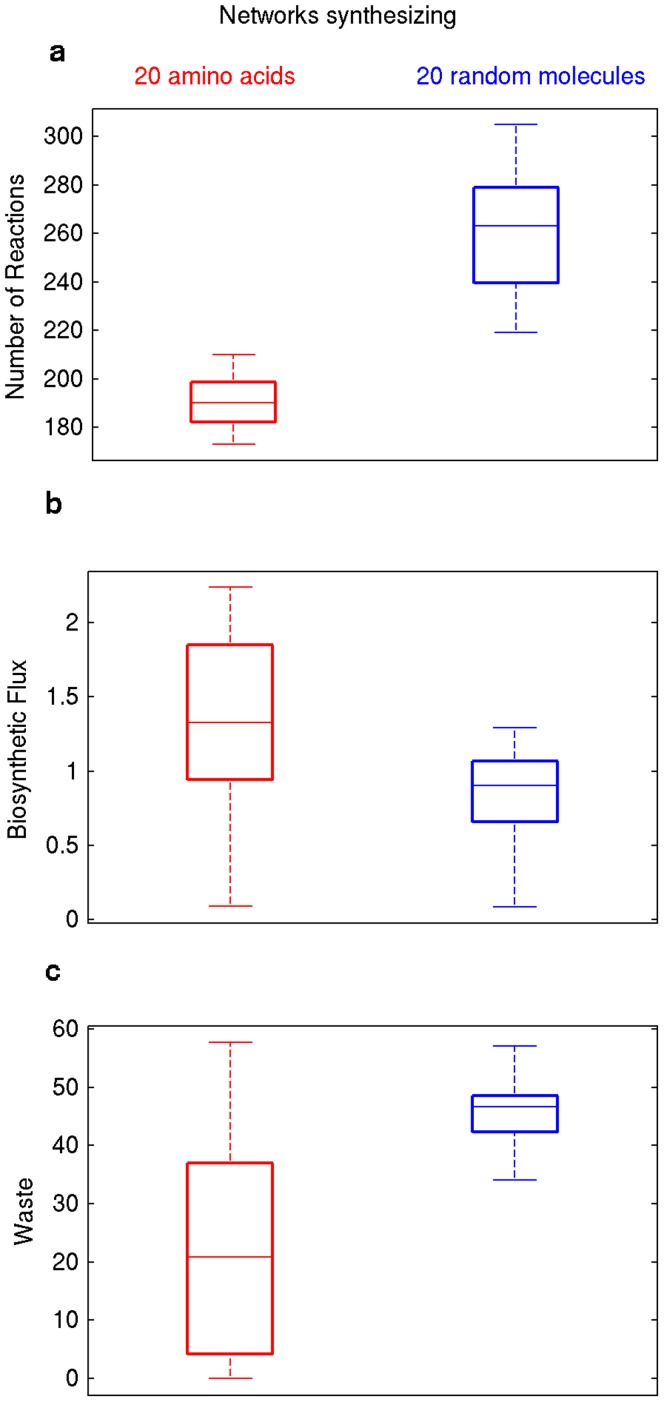
Synthesis of biochemically related compounds require less reactions, achieve higher biosynthetic flux with less waste. Box-plot of a) number of reactions, b) biosynthetic flux in mmoles per g DW hour, and c) waste in mmoles per g DW per hour in minimal networks synthesizing twenty amino acids (left panel) and synthesizing twenty random biomass molecules (right panel). Horizontal lines in the middle of each box mark the median. The edges of the boxes correspond to the 25th and 75th percentiles. Data is based on a sample of *n* = 80 for each box.

In our analysis of how required reaction numbers depend on a network’s biosynthetic abilities, we have purposely focused on randomly chosen biomass precursors, as they give us an unbiased view of this dependency. However, this relationship may change if one considers biochemically related biomass molecules. To obtain some insights how it changes, we next studied a group of molecules whose known biosynthesis pathways share several reactions. These are the 20 proteinaceous amino acids [58]. Figure 4a shows the number of reactions in minimal networks that can synthesize all 20 proteinaceous amino acids (left panel), as well as the number of reactions needed to synthesize 20 randomly chosen biomass molecules (right panel). Amino acid synthesizing networks need 191±10 (mean ± one standard deviation) reactions, 27 percent fewer than the 261±24 reactions needed to synthesize 20 arbitrary molecules. This difference is highly significant (P<10^−10^, Mann-Whitney U-test, n = 100) [59].

**Figure 5:**
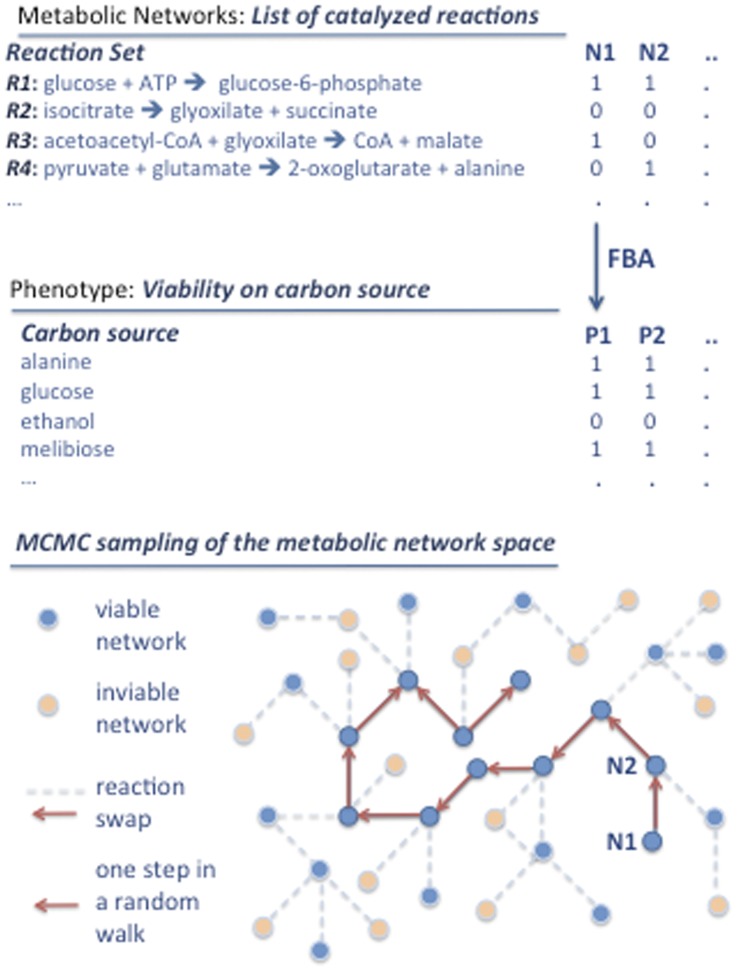
Exploration of a metabolic network space. Metabolic networks can be viewed as subsets of enzyme-catalyzed metabolic reactions in a global reaction set. Formally, they can be represented as binary vectors listing the reactions catalyzed by enzymes in an organism, as indicated for two hypothetical metabolic networks (N1, N2) in the figure. Metabolic phenotypes are computed from metabolic networks using FBA. They can be represented as binary vectors indicating the carbon sources (i.e.: alanine, glucose, melibiose,…) on which a network is viable, that is, on which it can synthesize a given set of (biomass) molecules. Neighboring networks (blue circles linked by edges) differ by a single reaction swap (edges between circles) that leaves the metabolic phenotype unchanged. A reaction swap consists of two changes: one random reaction addition (R4 in the example) and one random reaction deletion (R3 in the example). A series of successful reaction swaps is called a random walk (indicated by red arrows). The Markov Chain Monte Carlo (MCMC) technique allows one to randomly sample networks with a given phenotype by generating long random walks through genotype space, where each step in a walk consists of a reaction swap. The advantage of using reaction swaps is that they leave the number of reactions constant.

In minimal networks every reaction is active under the conditions we study. Our observations in the last paragraph thus also show that amino acid biosynthesis needs fewer active reactions than biosynthesis of arbitrary biomass molecules. One of our analyses above showed that fewer active reactions also imply higher biosynthetic flux, which raises the question whether the 20 amino acids can be synthesized at higher rates. The answer is yes (Figure 5b). Minimal networks that synthesize 20 arbitrary biomass molecules synthesize them 35 percent more slowly than minimal networks that synthesize 20 amino acids (P<10^−13^, *n* = 80, Mann-Whitney U-test) [59].

We next hypothesized that these dependencies might be explicable as a by-product of a lower cost of amino acid synthesis. If amino acids have fewer carbon molecules than the average biomass molecule, both fewer steps would be needed to synthesize them and a higher synthesis rate could be achieved. To test this hypothesis, we computed the average carbon content of all 20 amino acids, weighted by each amino acid’s stoichiometry in biomass, as 1.28±0.60 mmoles of carbon per mmole biomass. This molecular weight turned out not to be smaller but significantly larger than the carbon content of 20 random biomass molecules, also weighted by their stoichiometry in biomass (0.87±0.15 mmoles of carbon, P<2.2×10^−16^, one sample t-test, for *n* = 105 sets of 20 random biomass molecules). This means that low amino acid cost cannot explain a higher rate of amino acid production.

We next asked whether lower waste production might be responsible for the lower biosynthetic flux we observed. We therefore analyzed the quantity of secreted waste products in 80 networks that synthesize 20 amino acids, and in 80 networks that synthesize 20 random biomass compounds. Waste production is indeed significantly lower in networks that synthesize amino acids (P<10^−17^, Mann-Whitney U-test) [59]. Specifically, amino acid synthesizing networks produce 47 percent less waste than networks that synthesize arbitrary molecules. They excrete on average 22.8 mmoles of carbon waste per g DW hour (Figure 5c).

To provide a concrete example of a prominent waste molecule, consider the amount of secreted carbon dioxide. For each mole of carbon entering a network in the form of glucose, amino acid synthesizing networks excrete 0.18±0.12 (mean ± one standard deviation) moles of carbon per g DW hour as carbon dioxide, whereas networks synthesizing 20 arbitrary molecules excrete 0.31±0.06 mmoles of carbon per g DW hour as carbon dioxide (P<10^−25^, Mann-Whitney U-test) [59]. In sum, waste production is an important factor in explaining the smaller costs of amino acid biosynthesis compared to arbitrary biomass molecules.

## Discussion

We here studied typical properties of metabolic networks with recently developed techniques to create random and unbiased samples of metabolic networks from a vast space of such networks [46], [47]. Specifically, we studied the quantitative relationships between 6 different properties. These are the number of alternative sole carbon sources *N* that a network can use – its nutrient flexibility –, the number of (biomass) molecules *B* it can synthesize, the rate *S* at which it can synthesize these molecules, the number of reactions *R* in the network, the number of active reactions *R_A_*, that is, reactions that have nonzero metabolic flux, and the amount of waste *W* the network produces.

We focused first on how the number of minimally needed reactions *R* depends on *N* and *B*, because this number of reactions is an important design variable. Smaller networks would be easier to design and smaller genomes would be easier to synthesize [35]. More than that, biosynthetic processes are more controllable and predictable in a small metabolism [1], [18]. We found that for every additional molecule to be synthesized, a network needs on average three additional reactions.

In a related analysis, we focused on nutrient flexibility. The ability of a metabolic network to sustain life on multiple sources of chemical elements and energy is highly desirable in industrial production processes [31]. Examples include biofuel production from cellulosic biomass. Cellulosic biomass contains both hexoses (e.g., glucose) and pentoses, whose major constituent is xylose [31], [60]. Many yeast species, for instance, do not consume pentoses, and need to be engineered to have greater nutrient flexibility to make biofuel production more efficient [60]. In our analysis, we found that for every additional carbon source to be utilized, a network needs on average two additional reactions. Thus, it is more expensive, in terms of the number of needed reactions, to synthesize additional molecules than to synthesize the same molecules but from a larger number of alternative carbon sources.

Anecdotal evidence for the tendency that increased nutrient flexibility requires larger networks is provided by two networks for which nutrient flexibility is experimentally known [57], [61]. These are *E. coli* where *N* = 54 and *R* = 1396 and *Bacillus Subtilis* where *N* is smaller (*N* = 41) and so is *R* (*R* = 769). We also compared the computationally predicted number of carbon sources that can be utilized by metabolic models of seven organisms (Column 4 of Table 1). Although based on few data points, this analysis suggests that there is a significant correlation (Pearson’s r = 0.9, P = 0.005) between the *in silico* nutrient flexibilities and networks sizes of these model organisms.

**Table 1:** Biosynthetic ability *B*, number *R* of metabolic reactions (size of metabolic networks), number *R_A_* of number of active reactions, and number *N* of carbon sources predicted to be catabolized in computational metabolic models of various organisms.

Organism	*B*	*R*	*R_A_*	*N*	References
*Buchnera Aphidicola*	43	205	183	2	[97]
*Helicobacter pylori*	52	394	298	35	[98]
*Staphlococcus aureaus*	58	534	286	41	[99]
*Bacillus Subtilis*	59	769	327	97	[61]
*Methanosarcina barkeri*	63	531	352	6	[100]
*Escherichia coli*	67	1396	388	175	[57]
*Mycobacterium tubeculosis*	93	836	408	39	[101]

We took the number of synthesized molecules *B*, and the number of metabolic reactions *R* from the genome scale metabolic network reconstructions of these organisms, as listed in the column *‘References’*. Active reactions are reactions that have non-zero flux as determined by FBA. We computed nutrient flexibilities by testing the viability of metabolic models on all known carbon containing metabolites that are educts or products of reactions in the global network by FBA, and assumed a metabolite to be a carbon source if the metabolic model can utilize it.

As a result of dependencies between *N* and *R*, the minimally needed number of reactions varies widely among the networks we study. For example, for networks that can use 20 alternative carbon sources, it ranges from an average of 260 reactions (which would correspond to, for comparison, 19% of the whole *E. coli* network) needed to synthesize *B* = 20 molecules to an average of 381 reactions (29% of the *E. coli* network) to synthesize all *B* = 63 biomass molecules of *E. coli*. For networks that can use 80 alternative carbon sources, the largest number we studied, it increases from 398 (27%) to 518 reactions (37% of the *E. coli* network) as *B* increases from 20 to 63.

*N* and *B* can explain the vast majority (87%) of the variance in *R*, in a relationship that is close to linear. Note that the proportions in which biomass molecules are to be synthesized are immaterial, as long as the metabolic reactions to synthesize each required molecule are present. This is why the stoichiometry of biomass composition would not influence the relationships we observe. The remaining 13% of variance is unexplained by linear relationships among the variables we considered. Such unexplained variance could have multiple sources, and especially nonlinear relationships among the variables. For example, as the number of biomass molecules to be synthesized increases, fewer and fewer additional reactions might be required, because most precursors of the additional biomass molecules may already be synthesized by existing reactions.

We showed that the reactions needed to utilize an additional carbon-containing molecule or to synthesize an additional biomass molecule typically involve the additional molecule. For example, the utilization of an additional carbon source requires a reaction that catabolizes that molecule. The synthesis of an additional molecule requires a reaction that produces that molecule. We emphasize that the numbers of reactions required for an additional carbon source or biomass molecule reflect statistical patterns, averages over multiple networks. Some additional carbon sources or biomass molecules need more reactions than the average we identified, whereas others need fewer. (For some examples see Table S3, Table S4, Text S2 and Text S3.).

We also studied the number of active reactions – reactions with nonzero flux on a given carbon source. Quantitatively, we found that the number of active reactions increases by 3.6–4 reactions for each additional biomass molecule to be synthesized. Qualitatively, this tendency – increasing synthetic ability requires more active reactions – is not surprising, it is also corroborated by empirical data. Table 1 shows several well-known organisms with known metabolic network sizes *R* and biosynthetic abilities *B*. Note that, with exceptions, the number of active reactions *R_A_* tends to become higher as *B* increases for these organisms. Albeit based on few species, a regression analysis of the data in Table 1 shows that the relationship between *R_A_* and *B* is similar to what we find in our much larger samples of random networks (*R_A_* = 3.4*B*+120). Our approach has the advantage of allowing us to make quantitative statements about the number of active reactions required to synthesize additional molecules that are not just based on few, well studied organisms, as in Table 1, but on arbitrarily large and random samples of metabolic networks.

The identity of active metabolic reactions can depend strongly on the nutrient environment [12], [46], [62]. A reaction active in one environment may be inactive in another. Organisms would cope with such variation by regulating the activity of reactions, for example by regulating the expression of enzyme-coding genes or by regulating the enzymes themselves [63]. The importance of reaction activity in our analysis thus points towards the importance of regulating metabolic flux in response to the environment. Optimal regulation of enzymes is a key challenge in metabolic engineering [64], [65]. It will be an even greater challenge in a synthetic metabolism, especially if such a metabolism is to function *efficiently* in multiple chemical environments.

A second major set of analyses focused on how metabolic network properties influence biosynthetic flux *S*. These analyses are motivated by the fact that high synthesis rates of one or more target molecules are important goals of metabolic engineering [19]–[21]. Our first major observation in this regard concerns the number of reactions in a metabolic network. This number has virtually no influence on the attainable biosynthetic flux *S*. In stark contrast, the number of active reactions – reactions with nonzero flux – shows a strong negative association with biosynthetic flux. That is, the higher the biosynthetic flux is, the smaller is the number of active reactions. (This analysis is based on networks that have more than the minimally necessary number of reactions in a given environment. In minimal networks, all reactions must be active.) This observation is consistent with earlier work based on four microbial metabolic networks [12].

A second important influence on biosynthetic flux *S* is the number of biomass molecules to be synthesized. Specifically, every additional such molecule reduces *S* by approximately 5 percent. This influence is stronger than the influence of nutrient flexibility, where each additional carbon source changes biomass synthesis flux by 1 percent. That nutrient flexibility influences biosynthesis rates is known from experiment [31], [66]–[69]. For example, engineered yeast strains capable of growing on more carbon sources achieve higher product yields [31].

The amount of waste *W* that a network produces is a third, and the most important influence on biosynthetic flux *S*. Metabolic networks generally excrete waste, for example, in the form of carbon dioxide [12], acetate [12], [70], pyruvate [70] or glycerol [60], [66]. Not only does this mean wasted resources, some waste products may also be toxic and interfere with goals of metabolic engineering [27]. We found that biosynthetic flux *S* shows a strong negative correlation with waste production *W* in the form of excreted carbon. This observation is in qualitative agreement with experimental observations [60], [66], [70]. For example in *E. coli*, Weber *et al* [70] demonstrated that excretion of methylglyoxal, D- and L-lactate, pyruvate, and acetate decreases growth rates. We also observed that networks with higher nutrient flexibility produce less waste, such that for each additional carbon source, networks produce 0.1 mmoles less in excreted carbon waste. This relationship can help explain the positive influence of *N* on flux *S*. Experimental support for this observation exists as well. For example, Wisselink *et al*. [31] observed that increased ethanol production in engineered yeast with higher nutrient flexibility is caused by reduced production of the waste products xylitol and arabinitol. Relatedly, the elimination of glycerol formation increases the yield of ethanol up to 10 percent in yeast [66]. Ho *et al*. [60] report similar observations.

We also found that an analogous association does not exist for *B.* Networks seem to produce indistinguishable amounts of waste products regardless of how many biomass molecules they synthesize.

Overall, the quantities we examined can explain 84 percent of the variance in biosynthetic flux *S* we observed. This means, that these quantities account for most of the variation in the biosynthetic flux, and are thus important factors in the design of a synthetic metabolism.

In our final analysis, we showed that the nature of the molecules to be synthesized can influence biosynthetic flux. For example, in biotechnological applications, a metabolic network may need to synthesize molecules that are closely related, and whose biosynthetic pathways are therefore similar, in the sense that they share many reactions. Examples include vitamins and coenzymes [71], taxols and related taxoids [72], hydrocarbons and related ether lipids [73], as well as amino acids [74]. We examined the influence of biochemical relatedness by studying metabolic networks that synthesize all 20 amino acids, and by comparing them with networks that synthesize 20 arbitrary biomass molecules. In this analysis, we found that amino acids could be synthesized with fewer reactions, which reflects their biosynthetic relatedness. They can also be synthesized at a rate that is 35 percent higher, because their biosynthesis produces 47 percent less waste as excreted carbon. These differences are substantial, given that metabolic engineering in biotechnological processes typically increases synthesis rates of desired products by 5–10 percent [66], [75]–[77].

We conclude with some caveats and limitations of our analysis. First, we are well aware that there is still a gap between current metabolic engineering and synthetic biology experiments on the one hand, and theoretical studies such as ours on the other. We see the value of studies like ours as providing quantitative reference points for future experimental work in this area.

Second, flux balance analysis, on which our approach rests, assumes a metabolic system that is in a steady-state, such as might be achieved by a microbial population growing under a constant nutrient supply. This assumption ignores possible additional constraints that regulation and enzyme kinetics exert on a metabolism [78], [79]. While achieving proper regulation remains a big challenge in synthetic biology, we note that some of our observations are unlikely to be affected by this assumption. For example, relaxing this assumption would not reduce the minimal number of reactions needed for a given biosynthetic task.

Third, we focus on *typical* network properties, that is, properties of metabolic networks sampled at random from a large space of such networks. Optimization procedures that search metabolic networks space systematically may be able to identify individual networks whose properties deviate from those we identified as typical. For example, they may identify networks that are able to synthesize biomass with even fewer reactions or at higher rates. Such procedures have been successful in other combinatorial optimization problems. They include simulated annealing [80], [81], bi-level optimization [82], [83], OptFlux [84], convex optimization [85] and evolutionary optimization [86], [87]. The extent to which they can identify networks with superior design remains an important subject of future work.

Fourth, the expression of enzymes itself has a metabolic cost. This cost may be reduced by regulating enzyme expression depending on the nutrient environment (through a regulatory machinery whose expression may itself be costly.) We did not consider costs like these here, because they are poorly understood on a quantitative level. Their analysis also remains an important goal for future work.

Finally, we note that an organism’s genome encodes more than metabolism. Nonmetabolic genes, even in small genomes serve roles in systems that allow cell motility, signaling, secretion, and defense [14]. The heterogeneity of these systems will make general, quantitative statements about design constraints for entire genomes more difficult to obtain. In this regard, we note that the proportion of a gene’s genome devoted to metabolism increases in small genomes. For example, in the free-living *E. coli* with a large genome, fewer than 75 percent of genes exert metabolic functions [88]. In contrast, in some endosymbionts including *Buchnera*, *Wiggleswothia glossinidia* and *Thiomicrospira crunogena* more than 80 percent of genes are devoted to metabolic functions, and in yet others, such as *the gamma proteobacteria, Blochmanni floridanus* and *Wolbachia pipientis* more than 95 percent have metabolic functions [14]. Thus, the constraints we identified here would affect the majority and perhaps the vast majority of a synthetic minimal organism’s genome.

## Methods

The *metabolic genotype* of an organism comprises all genes that encode metabolic enzymes. This genotype can be compactly represented through a list of reactions that can take place in the organism and that are catalyzed by enzymes [36], [37], [89]–[92]. Each metabolic genotype exists in a vast metabolic *genotype space* that contains all possible metabolic networks – all possible combinations of reactions drawn from a set of biochemically feasible reactions – that *could* take place in a living system. According to our current knowledge, there are more than 5000 such reactions, which means that metabolic genotype space comprises 2^5000^ possible metabolic networks. The metabolic genotype or metabolic network of any one organism can be viewed as a point in this space. Two genotypes differing from each other in a single reaction are *neighbors* in this space. We refer to a network’s metabolic *phenotype* as the spectrum of chemical environments – defined by nutrients these environments contain – on which the network can synthesize a predetermined spectrum of molecules, as well as the rate at which it can synthesize these molecules. We call a network *viable* in a given environment if it can synthesize all these molecules in the environment.

### Flux Balance Analysis

Flux balance analysis (FBA) is a constraint-based modeling approach that predicts steady state metabolic fluxes – rates of substrate to product conversion – for all metabolic reactions in a metabolic network. It can thus also predict the biomass yield and other complex metabolic attributes of a metabolic network [78], [79]. Because FBA does not need kinetic information, but only stoichiometric information about the reactions involved, it is a widely used approach for analyzing the metabolism of well-studied organisms such as *E. coli* and *S. cerevisiae* [40], [78], [79]. The needed stoichiometric information comes from experimental biochemical analysis, as well as from comparative analyses of enzyme-coding genes in different, completely sequenced genomes. This information is encapsulated in a stoichiometric matrix **S** of dimensions *m×n*, where *m* denotes the number of metabolites, and *n* is the number of reactions in a network [40], [79]. FBA assumes that a metabolic network is in a metabolic steady-state, such as may occur in a microbial population that proliferates in an unchanging environment. Because mass needs to be conserved under these conditions, any vector **v** of metabolic fluxes – the rates at which a network’s metabolic reactions convert substrates into products – must satisfy the equation.
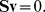



This equation typically has a large solution space of allowable fluxes. The size of this space can be reduced somewhat by placing biochemically motivated constraints on the irreversibility of some reactions and on maximal flux magnitudes [93]. In the (reduced) solution space, FBA then uses linear programming to identify regions in the space that maximize a quantity of interest, which can be represented by a linear objective function *Z* [40], [79]. More specifically, the linear programming formulation of an FBA problem can be written as:
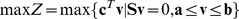
(1)where the vector **c** *^T^* stands for a transposed (^T^) array **c** of scalar coefficients that define the objective function. Vectors **a** and **b** contain lower and upper limits of reaction fluxes in the flux vector **v**, respectively. A particularly important quantity to be maximized is the rate of synthesis of a given set biomass molecules. We refer to this quantity as the *biosynthetic flux* (in units of mmol per g DW per hour) of a metabolic network. We used the software packages CPLEX (11.0, ILOG; http://www.ilog.com/) and CLP (1.4, Coin-OR; https://projects/coinor.org/Clp) to solve all linear programming problems that arise in this study.

### Biomass Molecules

The starting point of our analysis was a set of 67 biomass molecules from *E. coli* [57]. We used these molecules, because *E. coli’s* biomass composition is well studied, and many of its components – amino acids, nucleotides, etc. – also occur in most other free-living organisms. In addition, *E. coli* and its biomass molecules are highly relevant to biotechnological applications [18], [19], [21]. For some of our analyses, we needed to vary the spectrum of metabolites that a metabolic network needs to synthesize. Among the 67 *E. coli* biomass molecules, we allowed all but four molecules to vary. These four molecules are the “currency metabolites” adenosine 5′-diphosphate (ADP), phosphoric acid (Pi), pyrophosphoric acid (PPi), and hydrogen ions. A complete list of the biomass molecules we used can be found in Table S1.

### Carbon Sources

Any one metabolic network can only synthesize biomass if its chemical environment contains specific nutrients [94]. In FBA, these nutrients are represented by special exchange reactions that reflect transport of nutrients into the cell. The environments we study are minimal chemical environments that contain a single molecular source for each essential chemical element. These sources are oxygen, ammonium, inorganic phosphate, sulfate, sodium, potassium, cobalt, iron, protons, water, molybdate, copper, calcium, chloride, magnesium, manganese and zinc, as well as a single source of carbon. We study how variation in carbon sources constrains the composition of metabolic networks that needs to synthesize a given spectrum of molecules. We chose to vary carbon sources for this purpose, because of carbon’s centrality as a chemical element in biomass. A complete list of the carbon sources we used is given in the Table S2.

We computed the biomass growth of a network by taking the average of its biomass growth rates on each carbon source that the network is required to be viable on, except where mentioned otherwise.

### Global Network

Some of our analyses use a “global reaction network”. This global network contains a comprehensive set of known biochemical reactions from multiple organisms, and it has universal biosynthetic abilities. We are fully aware that no such network is likely to exist in any one organism. We use this global network merely as a starting point for successive elimination of reactions, as described in the next section. We generated this global network by merging reactions from two sources, as described in more detail in [47]. The first is the LIGAND reaction database of the Kyoto Encyclopedia of Genes and Genomes (KEGG) [36], [37], [89]–[92]. The second is the complete metabolic reaction set of *E. coli* (iAF1260), which contains 1397 non-transport reactions [57]. We excluded from this network (i) all reactions involving polymer metabolites of unspecified numbers of monomer units, (ii) general polymerization reactions with uncertain stoichiometry, (ii) reactions involving glycans, due to their complex structure, (iv) reactions with unbalanced stoichiometry, and (v) reactions involving complex metabolites without chemical information about their structure [47]. After these procedures the global network contained 5906 internal (non-transport reactions) and 5030 metabolites.

### Essential Reactions and Minimal Networks

A gene whose deletion abolishes the viability of an organism is called an *essential gene*. Analogously, an *essential reaction* in a metabolic network is a reaction that cannot be removed without abolishing the organism’s viability in a given environment. A *minimal* metabolic network is a network from which not a single reaction can be removed without abolishing viability in a given environment. In other words, all reactions of a minimal network are essential in that environment. We emphasize that a minimal network is *not* the smallest possible viable network, which would be difficult to find in a vast space of metabolic networks. We note that there may be multiple minimal networks, which contain different pathways among a set of possible alternate pathway from a nutrient to a biomass precursor. These networks need not have the same size. We also note that the number of essential reactions in any one non-minimal network may be smaller than the size of a minimal network, because non-minimal networks may contain alternate pathways able to by-pass any one reaction.

### Generation of Minimal Networks

To analyze the smallest number of reactions that a network needs to have in order to (i) synthesize a given number *B* of biomass molecules on (ii) each of *N* different carbon sources, we created and analyzed many minimal networks with different values of *B* and *N*. Because there are astronomically many combinations of different values of *B* and *N*, and because the FBA approach we use is computationally expensive, we focused on subsets of such combinations, which we created as follows.

First, we created 10 different sets each of *B* = 40, *B* = 30, and *B* = 20 randomly chosen biomass molecules from the set of 63 *E. coli* biomass molecules (excluding the four *‘currency’* metabolites mentioned above in the Section ‘Biomass Molecules’, which are found in all of the sets) [57]. These sets served as the basis for our analysis of networks that vary in their biosynthetic ability *B*. To arrive at identical sample sizes for subsequent analyses, we also included 10 “sets” with B = 63, that is, each of these sets contained all 63 biomass molecules. In total, we thus created a total of 40 (10×4) sets of biomass molecules.

Second, for each of these 40 sets we determined 4 different sets of nutrients, where each set contained a different number of randomly chosen carbon sources from the list of carbon sources we used (see Section Carbon Sources). Specifically, these sets of nutrients contained *N* = 20, *N* = 40, *N* = 60, and *N* = 80 carbon sources. Thus, up to this second step we had generated a total of 160 (40×4) combinations of sets of biomass molecules and nutrients.

Finally, for each of these 160 combinations, we created 5 minimal metabolic networks. To create each minimal network, we used the following procedure. We started from the global network and sequentially removed individual randomly chosen reactions from it. After each reaction removal we verified that the network was still viable – able to synthesize all *B* biomass molecules in the set – when each of the *N* nutrients was provided as the sole carbon source, that is, the network was required to be viable on each carbon source. If that was not the case, we reversed the reaction elimination and removed a different, randomly chosen reaction, until the resulting network was viable. We continued this procedure until no further reactions could be removed from the network without abolishing viability. In this fashion, we generated 800 (160×5) minimal networks. Note that carrying out this procedure repeatedly may not arrive at the same minimal network, because reactions are removed at random. That is, different minimal networks may contain different numbers and different sets of reactions. What unites them is that all their reactions are essential.

### Minimal Networks with Isostoichiometric Biomass Composition

We asked whether the stoichiometric composition of biomass, that is, the relative amounts of different biomass molecule in biomass, influences the relationships we explore here. To this end, we generated minimal networks synthesizing given biomass compounds, as described in the previous section, with the difference that these networks synthesized these compounds in equal molarities, that is isostoichiometrically. In other words, for the purpose of this analysis we changed the stoichiometric coefficients ***c*** ^T^ in the biomass growth function of Equation 1 to a value of one. We used the same combinations of sets nutrients and biomass molecules as described above, except that we generated only 2 minimal networks (instead of 5) for each combination, in order to reduce computational cost. In total, we thus analyzed 320 minimal networks with isostoichiometric biomass composition.

### Minimal Networks Synthesizing 20 Amino Acids

To study examples of networks that synthesize biochemically related biomass molecules, we studied minimal networks synthesizing only the 20 proteinaceous amino acids. We generated these networks as described in ‘Generation of Minimal Networks’, except that we did not choose the biomass molecules to be synthesized at random. More specifically, we created 10 sets each of *N* = 20, *N* = 40, *N* = 60, and *N* = 80 randomly chosen carbon sources, and analyzed 8 minimal networks for each set, for a total of 320 networks.

### MCMC Sampling and Random Networks

As we mentioned earlier, changing the genotype of a network does not necessarily cause a change in its phenotype. One can take advantage of this property to generate arbitrarily large and unbiased random samples of metabolic networks with any desired property (see Figure 5), such that viable networks with a given number of reactions are sampled uniformly from the space of such networks. Such samples are central to our analysis. To create them we used a procedure built on Markov Chain Monte Carlo (MCMC) sampling described previously [46], [47]. This procedure fulfills the important detailed balance condition for MCMC sampling. Briefly, the procedure constructs a sequence of metabolic networks, where the next network in the sequence is created from the previous network through a reaction swap. Such a reaction swap consists of the removal of a randomly chosen reaction from the network, followed by the addition of a randomly chosen reaction taken from the global network. We used such reaction swaps, because they preserve the exact number of reactions in a metabolic network, which is important for our analysis. If a reaction swap preserves viability of the network in a given environment, then the swap is accepted, otherwise it is rejected, and a new swap is attempted until a viable genotype is found. We note that subsequent networks in a sequence show autocorrelation in their properties, and are thus not suitable for random sampling. Past work has shown that after 5×10^3^ swaps, the autocorrelation of two genotypes becomes negligible [46]. We therefore started the random walk from *E. coli* metabolic network and after 2.5×10^6^ reaction swaps, we sampled networks every 5×10^3^ steps in a sequence. Overall, the network samples we created comprise 1000 networks for each condition we study. We did not subject transport reactions to the reaction swap procedure.

To compare our observations from randomly sampled metabolic networks to a reference from biology, it is useful to use a network from a well-studied organism. For this purpose, we used the metabolic network of *E. coli*, because *E. coli* is able to survive in multiple different environments [95], [96].

### Generation of Starting Networks for MCMC Sampling

To initiate the MCMC procedure that generates random samples of networks with a given set of properties, we needed starting networks that have these properties. Specifically, we needed to create networks with a given number of reactions *R*, nutrient flexibility *N*, and biosynthetic ability *B*. To this end, we started with the same 160 sets of *N* nutrients and *B* biomass molecules described in ‘Generation of Minimal Networks’. We created for each set 5 viable networks that differ in their number of reactions, that is, they had 400, 500, 600, 700 and 800 reactions. To create these networks, we used the same procedure as for the production of minimal networks, except that we stopped removing reactions when a network with the desired number of reactions had been reached. At the end of this procedure, we had 800 networks (160×5) with various combinations of the 3 network properties. We used each of these networks as starting networks for MCMC sampling, and generated 20 random viable networks from each of the 800 starting networks, for a total of 16000 random networks. This amounts to 200 networks for each different combination of *N*, *B*, and *R*.

We performed all our analyses using MATLAB (7.10.0, The MathWorks Inc., Natick, MA, R2010a) and R (R Development Core Team, 2008).

## Supporting Information

Figure S1 **Biomass stoichiometry does not affect the number of reactions in a minimal network.** The vertical axis shows the number of reactions in minimal networks as a function of a) biosynthetic ability and b) nutrient flexibility. Dots and lengths of error bars correspond to means and one standard deviation. Each blue dot of size 200 indicates networks with the biomass stoichiometry of E. coli [57]. Each red dot of size 80 indicates networks with isostoichiometric biomass (see Methods). Red and blue means do not differ from each other significantly (P>0.40, Mann-Whitney U-test).
(TIF)

Figure S2 **Biosynthetic flux distribution.** The flux distributions in units of mmoles per g DW per hour are shown for each combination of biosynthetic ability (B = 20 first row, B = 30 second row, B = 40 third row, B = 63 last row) and nutrient flexibility (N = 20 first column, N = 30 second column, N = 40 third column, N = 63 last column) that we examined. Data are based on 16,000 random viable networks, as described in methods (1000 networks per panel).
(TIF)

Figure S3 **Distribution of number R of reactions.** The distributions of R are shown for each combination of biosynthetic ability (B = 20 first row, B = 30 second row, B = 40 third row, B = 63 last row) and nutrient flexibility (N = 20 first column, N = 30 second column, N = 40 third column, N = 63 last column) that we examined, and for in total 800 minimal viable networks (50 Networks per panel).
(TIF)

Table S1 List of E. coli’s Biomass Compounds [57].
(DOC)

Table S2 List of carbon sources used in this study.
(DOC)

Table S3 Examples of reactions required to synthesize additional biomass molecules. The table contains 20 arbitrary biomass molecules (left), and a list of reactions that are required to synthesize the molecule in a random minimal network (in addition to the reactions that the network needed to synthesize other biomass molecules). The analysis is based on minimal networks that are required (i) to synthesize 62 E. coli biomass molecules and (ii) to be viable on glucose. The Table illustrates that the number of additional reactions needed depends on the biomass molecule. (It may also depend on other reactions in a network, but for each biomass molecule results from only one network are shown.)
(DOC)

Table S4 Examples of reactions required to utilize additional carbon sources. The table contains arbitrary carbon sources (left) and a list of reactions that are required to utilize the carbon source in a random minimal network (in addition to the reactions that the network needs to utilize other carbon sources). The analysis is based on minimal networks that were required (i) to synthesize all E. coli biomass molecules and (ii) to be viable on 30 other carbon sources. The Table illustrates that the number of additional reactions needed depends on the carbon source. (It may also depend on other reactions in a network, but for each carbon source results for only one network are shown.)
(DOC)

Text S1 Confidence Interval Calculation.
(DOC)

Text S2 Examples of reactions needed to metabolize new carbon sources.
(DOC)

Text S3 Examples of reactions needed to synthesize additional biomass molecules.
(DOC)
