# Supplementary figures and images for: Design Constraints on a Synthetic Metabolism
Source: PLoS One. 2012 Jun 29;7(6):e39903. doi: 10.1371/journal.pone.0039903 (PMC3387219; doi:10.1371/journal.pone.0039903)

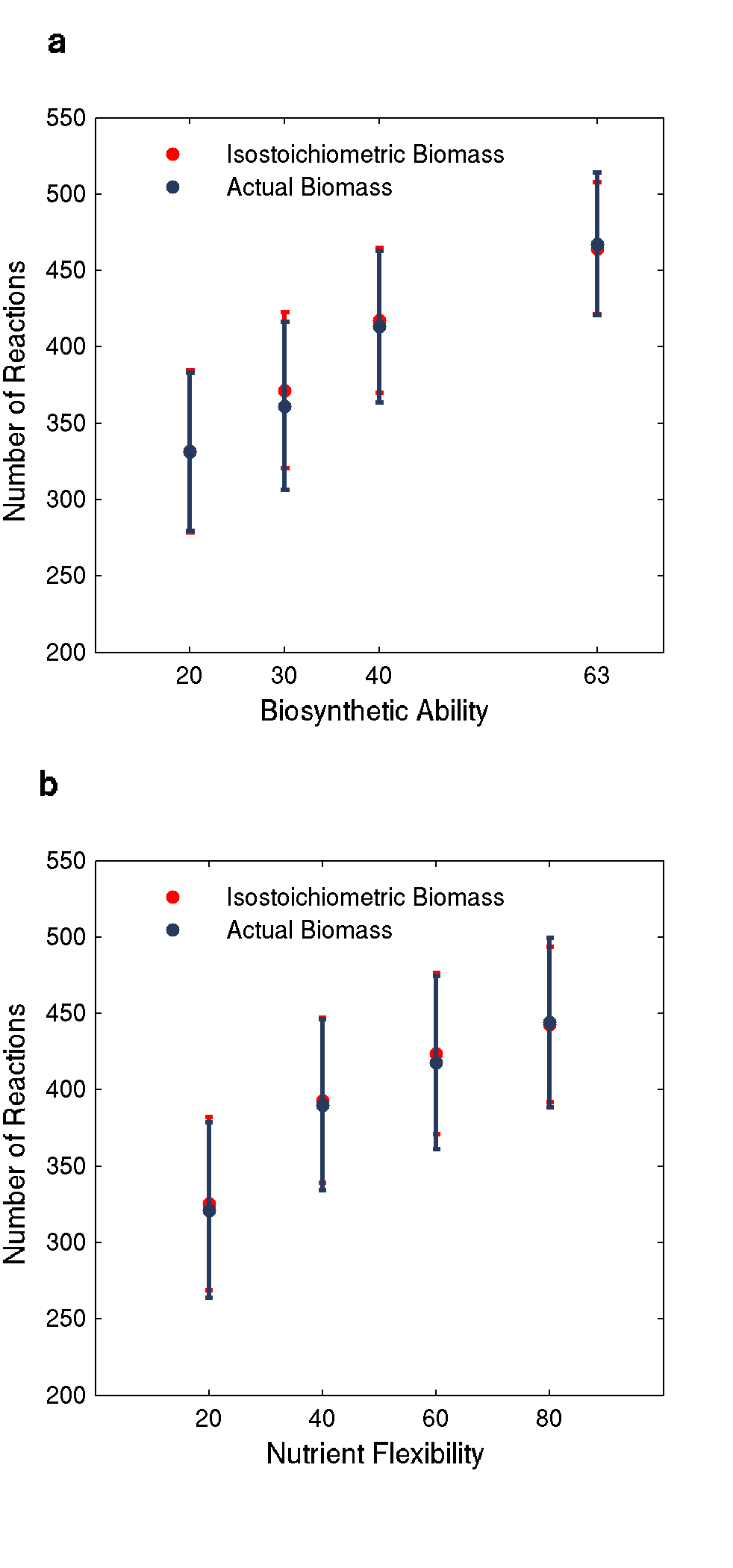

Supplement: Figure S1 — Biomass stoichiometry does not affect the number of reactions in a minimal network. The vertical axis shows the number of reactions in minimal networks as a function of a) biosynthetic ability and b) nutrient flexibility. Dots and lengths of error bars correspond to means and one standard deviation. Each blue dot of size 200 indicates networks with the biomass stoichiometry of E. coli [57]. Each red dot of size 80 indicates networks with isostoichiometric biomass (see Methods). Red and blue means do not differ from each other significantly (P>0.40, Mann-Whitney U-test). (TIF) [file pone.0039903.s001.tif]

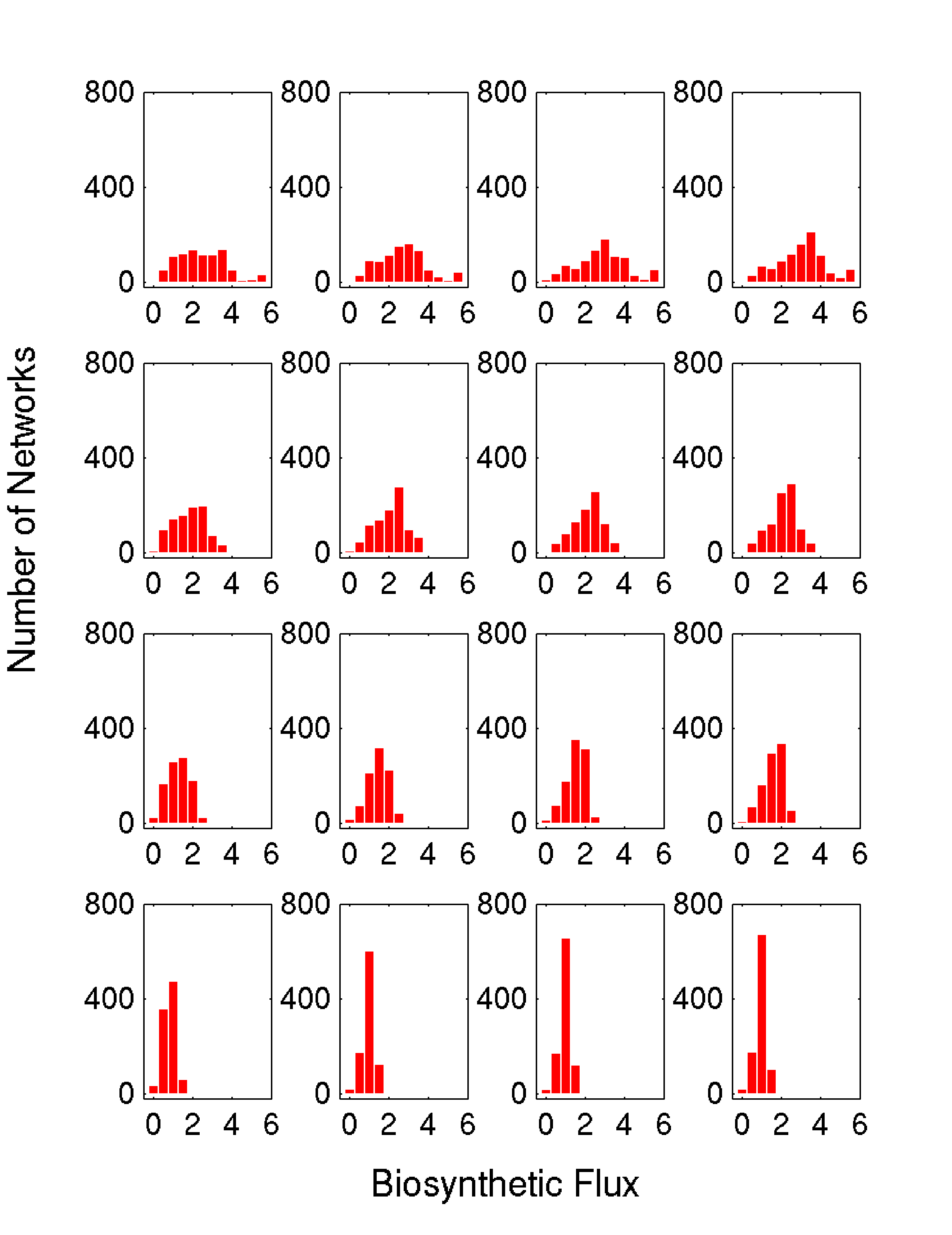

Supplement: Figure S2 — Biosynthetic flux distribution. The flux distributions in units of mmoles per g DW per hour are shown for each combination of biosynthetic ability (B = 20 first row, B = 30 second row, B = 40 third row, B = 63 last row) and nutrient flexibility (N = 20 first column, N = 30 second column, N = 40 third column, N = 63 last column) that we examined. Data are based on 16,000 random viable networks, as described in methods (1000 networks per panel). (TIF) [file pone.0039903.s002.tif]

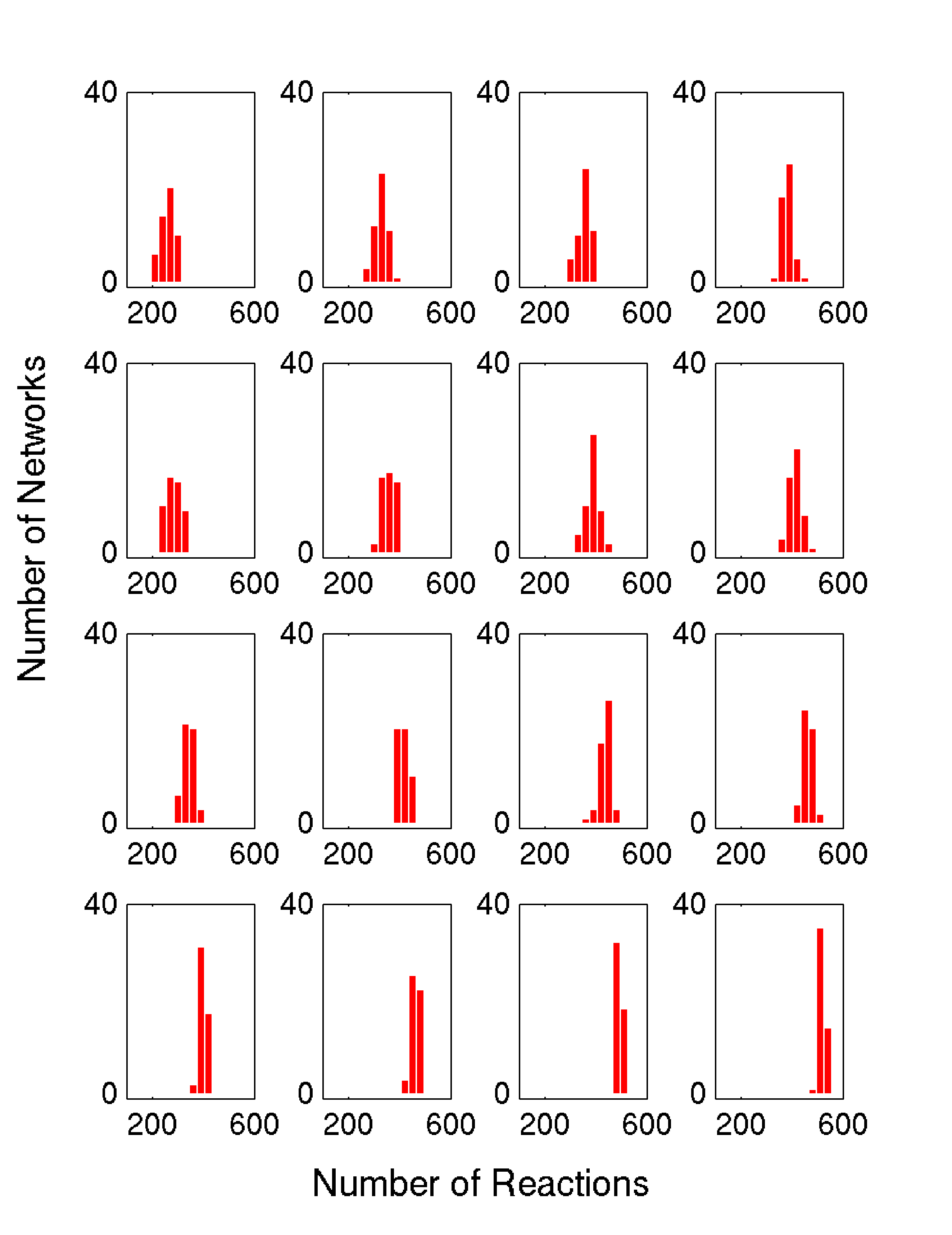

Supplement: Figure S3 — Distribution of number R of reactions. The distributions of R are shown for each combination of biosynthetic ability (B = 20 first row, B = 30 second row, B = 40 third row, B = 63 last row) and nutrient flexibility (N = 20 first column, N = 30 second column, N = 40 third column, N = 63 last column) that we examined, and for in total 800 minimal viable networks (50 Networks per panel). (TIF) [file pone.0039903.s003.tif]
