# Supplementary material for: Design Constraints on a Synthetic Metabolism
Source: PLoS One. 2012 Jun 29;7(6):e39903. doi: 10.1371/journal.pone.0039903 (PMC3387219; doi:10.1371/journal.pone.0039903)
Supplement: Table S1 — List of E. coli’s Biomass Compounds [57]. (DOC) [file pone.0039903.s004.doc]

| L-alanine |  |
| --- | --- |
| L-arginine | potassium |
| L-asparagine | ammonium |
| L-aspartate | magnesium |
| L-cysteine | calcium |
| L-glutamine | reduced iron |
| L-glutamate | iron trication |
| glycine | copper |
| L-histidine | manganese |
| L-isoleucine | molybdenum |
| L-leucine | cobalt |
| L-lysine | zinc |
| L-methionine | chloride |
| L-phenylalanine | sulfate |
| L-proline | water |
| L-serine | coenzyme-A |
| L-threonine | NAD |
| L-tryptophan | NADP |
| L-tyrosine | FAD |
| L-valine | 5,6,7,8-tetrahydrofolate |
| datp | 5,10-methylenetetrahydrofolate |
| dttp | 10-formyltetrahydrofolate |
| dgtp | thiamine diphosphate |
| dctp | pyridoxal 5'-phosphate |
| CTP | protoheme |
| GTP | siroheme |
| UTP | undecaprenyl diphosphate |
| ATP | S-sdenosyl-L-methionine |
| murein disaccharide | 2-octaprenyl-6-hydroxyphenol |
| KDO(2)-lipid IV(A) | riboflavin |
| Phosphatidylethanolamine (dihexadecanoyl, n-C16:0) | phosphatidylethanolamine (dihexadecanoyl, n-C16:1) |
| Phosphatidylethanolamine phosphate (dihexadecanoyl, n-C16:0) | phosphatidylethanolamine phosphate (dihexadecanoyl, n-C16:1) |
