# Supplementary material for: Design Constraints on a Synthetic Metabolism
Source: PLoS One. 2012 Jun 29;7(6):e39903. doi: 10.1371/journal.pone.0039903 (PMC3387219; doi:10.1371/journal.pone.0039903)
Supplement: Table S2 — List of carbon sources used in this study. (DOC) [file pone.0039903.s005.doc]

| 1,4-alpha-D-glucan | UMP | Lactose |
| --- | --- | --- |
| 3-hydroxycinnamic | Glycerol | Butyrate |
| 3-(3-hydroxy-phenyl)propionate | alpha-D-Ribose | Hexadecanoate |
| Phosphate | D-Ribose | D-Lactate |
| AMP | Fumarate | D-Gluconate |
| Pyruvate | D-Galactose | L-Arabinose |
| L-Glutamate | IMP | Hypoxanthine |
| 2-Oxoglutarate | D-Alanine | N-Acetylneuraminate |
| UDPglucose | Putrescine | D-Mannose |
| D-Glucose | N-Acetyl-D-glucosamine | Inosine |
| Acetate | GMP | Uridine |
| Glycine | Adenine | L-Xylulose |
| L-Alanine | L-Proline | D-Glucosamine |
| Succinate | L-Malate | Deoxyguanosine |
| UDP-N-acetyl-D-glucosamine | L-Asparagine | D-Galacturonate |
| L-Aspartate | Citrate | 4-Aminobutanoate |
| Reduced | D-Mannose | D-Glucosamine |
| UDPgalactose | Glycolate | dAMP |
| CMP | Propionate | dGMP |
| Formate | Acetoacetate | dTMP |
| Sulfate | UDP-D-glucuronate | dUMP |
| L-Arginine | Agmatine | Xanthine |
| L-Glutamine | D-Xylose | Guanosine |
| L-Serine | Dihydroxyacetone | D-Mannitol |
| Formaldehyde | L-Lactate | alpha-D-Galactose |
| L-Ascorbate | L-Threonine | Ethanol |
| L-Tryptophan | Ethanolamine | Cytidine |
| Acetaldehyde | D-Glucuronate | Propanal |
| D-Fructose | UDP-N-acetyl-D-galactosamine | D-Malate |
| Sucrose | 2-Dehydro-3-deoxy-D-gluconate | L-Rhamnose |
| D-Glucose | Maltose | Deoxyuridine |
| Glycerol | Adenosine | Deoxyadenosine |
| D-Fructose | Thymidine | D-Glyceraldehyde |
| L-Cysteine | dCMP | N-Acetyl-D-mannosamine |
| D-Glucose | Guanine | Xanthosine |
| sn-Glycero-3-phosphocholine | 2',3'-Cyclic | octadecanoate |
| D-Serine | 2',3'-Cyclic | Allantoin |
| L-Idonate | Dodecanoate | Decanoate |
| D-Cysteine | N-Acetylmuramate | Hexanoate |
| D-Sorbitol | Glycerol | Ornithine |
| D-Glucarate | Glycerophosphoglycerol | Galactitol |
| D-Galactarate | N-Acetyl-D-glucosamine | Xanthosine |
| D-Galactonate | D-Glucuronate | Maltotriose |
| Deoxycytidine | Melibiose | Maltohexaose |
| L-tartrate | Deoxyinosine | Maltotetraose |
| D-Fructuronate | Phenylpropanoate | 2',3'-Cyclic |
| D-Alanyl-D-alanine | 3'-cmp | butanesulfonate |
| O-Phospho-L-serine | 3'-GMP | ethanesulfonate |
| L-Fucose | 2',3'-Cyclic | fructoselysine |
| 5-Dehydro-D-gluconate | dIMP | Glycerophosphoserine |
| Trehalose | Fe(III)dicitrate | Galactonate |
| sn-Glycero-3-phospho-1-inositol | 2,3-diaminopropionate | Hexadecenoate |
| sn-Glycero-3-phosphoethanolamine | octanoate | Maltopentaose |
| Ammonium | tetradecanoate | octadecenoate |
| 3'-AMP | 2(alpha-D-Mannosyl)-D-glycerate | L-Prolinylglycine |
| 3'-UMP | L-Threonine | psicoselysine |
| Cys-Gly | L-Lyxose | tetradecenoate |
| L-alanine-D-glutamate-meso-2,6-diaminoheptanedioate | N-Acetyl-D-glucosamine(anhydrous)N-Acetylmuramic | L-alanine-D-glutamate-meso-2,6-diaminoheptanedioate-D-alanine |
| D-Allose |  |  |
