# Supplementary material for: Design Constraints on a Synthetic Metabolism
Source: PLoS One. 2012 Jun 29;7(6):e39903. doi: 10.1371/journal.pone.0039903 (PMC3387219; doi:10.1371/journal.pone.0039903)
Supplement: Table S3 — Examples of reactions required to synthesize additional biomass molecules. The table contains 20 arbitrary biomass molecules (left), and a list of reactions that are required to synthesize the molecule in a random minimal network (in addition to the reactions that the network needed to synthesize other biomass molecules). The analysis is based on minimal networks that are required (i) to synthesize 62 E. coli biomass molecules and (ii) to be viable on glucose. The Table illustrates that the number of additional reactions needed depends on the biomass molecule. (It may also depend on other reactions in a network, but for each biomass molecule results from only one network are shown.) (DOC) [file pone.0039903.s006.doc]

| **Biomass Molecule** | **Required Additional Reactions** |
| --- | --- |
| 2-octaprenyl-6-hydroxyphenol | 2-octaprenylphenol hydroxylase, octaprenyl-hydroxybenzoate decarboxylase, hydroxybenzoate octaprenyltransferase, chorismate pyruvate lyase, octaprenyl pyrophosphate synthase |
| arginine | N-acetylglutamate synthase, N-acetyl-g-glutamyl-phosphate reductase, acetylglutamate kinase, acetylornithine transaminase, acetylornithine deacetylase, argininosuccinate lyase, argininosuccinate synthase, ornithine carbamoyltransferase |
| asparagine | asparagine synthase |
| coenzyme A | 2-dehydropantoate 2-reductase, 3-methyl-2-oxobutanoate hydroxymethyltransferase, phosphopantothenate-cysteine ligase, phosphopantothenoylcysteine decarboxylase, pantothenate synthase, aspartate 1-decarboxylase, dephospho-CoA kinase, pantetheine-phosphate adenylyltransferase, pantothenate kinase |
| dATP | ribonucleoside-triphosphate reductase (ATP) |
| dCTP | ribonucleoside-triphosphate reductase (CTP) |
| dGTP | nucleoside-diphosphate kinase (ATP:dGDP), ribonucleoside-diphosphate reductase (GDP) |
| dTTP | nucleoside-diphosphate kinase (ATP:dTDP), dTMP kinase, uridylate kinase (dUMP), thymidylate synthase, ribonucleoside-diphosphate reductase (UDP) |
| FAD | FMN adenylyltransferase, riboflavin kinase |
| histidine | ATP phosphoribosyltransferase, imidazoleglycerol-phosphate dehydratase, imidazole-glycerol-3-phosphate synthase, histidinol-phosphate transaminase, histidinol-phosphatase, histidinol dehydrogenase, phosphoribosyl-AMP cyclohydrolase, phosphoribosyl-ATP pyrophosphatase, 1-imidazole-4-carboxamide isomerase |
| isoleucine | dihydroxy-acid dehydratase, ketol-acid reductoisomerase, 2-aceto-2-hydroxybutanoate synthase, isoleucine transaminase, L-threonine deaminase |
| KDO(2)-lipid IV(A) | UDP-3-O-glucosamine acyltransferase, UDP-N-acetylglucosamine acyltransferase, 3-deoxy -D-manno-octulosonic -acid 8-phosphate, tetraacyldisaccharide 4'kinase, 3-deoxy-D-manno-octulosonic acid transferase, 3-deoxy-manno-octulosonate cytidylyltransferase, 3-deoxy-manno-octulosonate-8-phosphatase, UDP-sugar hydrolase, UDP-3-O-acetylglucosamine deacetylase, lipid A disaccaride synthase, arabinose-5-phosphate isomerase |
| leucine | 2-isopropylmalate hydratase, 3-isopropylmalate dehydrogenase, 3-isopropylmalate dehydratase, 2-Oxo-4-methyl-3-carboxypentanoate decarboxylation, 2-isopropylmalate synthase, leucine transaminase |
| lysine | diaminopimelate decarboxylase |
| acetyl-CoA | 2-octaprenylphenol hydroxylase, octaprenyl-hydroxybenzoate decarboxylase, hydroxybenzoate octaprenyltransferase, chorismate pyruvate lyase, octaprenyl pyrophosphate synthase |
| alanine | phosphogluconate dehydrogenase |
| aspartate | malic enzyme |
| chorismate | 2-dehydropantoate 2-reductase, 3-methyl-2-oxobutanoate hydroxymethyltransferase, phosphopantothenate-cysteine ligase, phosphopantothenoylcysteine decarboxylase, pantothenate synthase, aspartate 1-decarboxylase, dephospho-CoA kinase, pantetheine-phosphate adenylyltransferase, pantothenate kinase |
| protoheme | Valine-pyruvate aminotransferase |
| putrescine | Undecaprenyl diphosphate synthase |
