# Supplementary material for: Design Constraints on a Synthetic Metabolism
Source: PLoS One. 2012 Jun 29;7(6):e39903. doi: 10.1371/journal.pone.0039903 (PMC3387219; doi:10.1371/journal.pone.0039903)
Supplement: Table S4 — Examples of reactions required to utilize additional carbon sources. The table contains arbitrary carbon sources (left) and a list of reactions that are required to utilize the carbon source in a random minimal network (in addition to the reactions that the network needs to utilize other carbon sources). The analysis is based on minimal networks that were required (i) to synthesize all E. coli biomass molecules and (ii) to be viable on 30 other carbon sources. The Table illustrates that the number of additional reactions needed depends on the carbon source. (It may also depend on other reactions in a network, but for each carbon source results for only one network are shown.) (DOC) [file pone.0039903.s007.doc]

| **Carbon Source** | **Required Additional Reactions** |
| --- | --- |
| D-galactose | galactokinase, UDPglucose--hexose-1-phosphate uridylyltransferase, UDPglucose 4-epimerase |
| glycerophosphoserine | glycerophosphodiester phosphodiesterase |
| 1,4-alpha-D-glucan | maltodextrin glucosidase |
| 2(alpha-D-Mannosyl)-D-glycerate | 2(alpha-D-Mannosyl-6-phosphate)-D-glycerate hydrolase |
| L-ascorbate | 3-keto-L-gulonate 6-phosphate decarboxylase, L-ribulose-phosphate 4-epimerase, L-xylulose 5-phosphate 3-epimerase |
| agmatine | agmatinase |
| IMP | 5'-nucleotidase |
| dAMP | deoxyadenosine deaminase, purine-nucleoside phosphorylase |
| L-tartrate | L(+)-tartrate dehydratase |
| deoxyguanosine | purine-nucleoside phosphorylase (Deoxyguanosine) |
| 2',3'-Cyclic | 2',3'-Cyclic UMP phasphatase |
| phenylpropanoate | 4-hydroxy-2-oxopentanoate aldolase, 2,3-dihydroxyphenylpropionate dehydrogenase, diaminohydroxyphosphoribosylaminopryrimidine deaminase, 2,3-dihydroxypheylpropionate 1,2-dioxygenase, phenylpropanoate Dioxygenase, 2,3-dihydroxypheylpropionate 1,2-dioxygenase, 2-hydroxy-6-ketonona-2,4-dienedioic acid hydrolase, 2-oxopent-4-enoate hydratase |
| glycerophosphoglycerol | glycerophosphodiester phosphodiesterase |
| L-idonate | L-idonate 5-dehydrogenase |
| D-ribose | ribokinase |
| lactose | b-galactosidase |
| D-galactarate | 5-dehydro-4-deoxyglucarate aldolase, galactarate dehydratase |
| hypoxanthine | ureidoglycolate hydrolase, malate synthase, allantoinase, 5'-nucleotidase (GMP), purine-nucleoside phosphorylase (Guanosine), guanine deaminase, purine-nucleoside phosphorylase (Inosine), L-serine deaminase |
| N-acetyl-D-glucosamine 1-phosphate | glycerophosphodiester phosphodiesterase |
| asparagine | asparaginase |
