# Supplementary material for: Design Constraints on a Synthetic Metabolism
Source: PLoS One. 2012 Jun 29;7(6):e39903. doi: 10.1371/journal.pone.0039903 (PMC3387219; doi:10.1371/journal.pone.0039903)
Supplement: Text S1 — Confidence Interval Calculation. (DOC) [file pone.0039903.s008.doc]

**Confidence Interval Calculation**

We used the Matlab Statistical Package to calculate confidence intervals throughout the paper. The function makes use of the expression below to calculate 95% confidence limits for means.

$P\left\{\overline{Y}-\frac{1.96\sigma}{\sqrt{n}}\leq\mu\leq\overline{Y}\text{+}\frac{1.96\sigma}{\sqrt{n}}\right\}=1-\alpha$,

where *P* is the probability that an actual mean *μ* lies in the indicated interval,
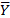
 is the sample mean, *σ* is the sample variance, and *n* is the sample size. The value of α is equal to 0.05, for we calculated 95% confidence intervals of mean.

To calculate 95% confidence limits for variances, we made use of the expression

$P\left\{\frac{(n-1)s^{2}}{X_{(\frac{\alpha}{2})\left[n-1\right]}^{2}}\leq\sigma^{2}\leq\frac{(n-1)s^{2}}{X_{(1-(\frac{\alpha}{2}))\left[n-1\right]}^{2}}\right\}=1-\alpha$,

where *P* is the probability that the actual variance σ^2^ lies in the indicated interval, *n* is the sample size, *s^2^* is the sample variance, and *X^2^* is the value of the chi square distribution with *n*-1 degrees of freedom at a value α/2 (for the left argument). The value of α is equal to 0.05, for we calculated 95% confidence intervals of variance.
