# Supplementary material for: Design Constraints on a Synthetic Metabolism
Source: PLoS One. 2012 Jun 29;7(6):e39903. doi: 10.1371/journal.pone.0039903 (PMC3387219; doi:10.1371/journal.pone.0039903)
Supplement: Text S2 — Examples of reactions needed to metabolize new carbon sources. (DOC) [file pone.0039903.s009.doc]

**Examples of reactions needed to metabolize new carbon sources**

These examples take a network size reduction approach to illustrate the kinds of reactions needed to metabolize new carbon sources.

**Example 1.** We generated a minimal network that was required to synthesize *B*=63 biomass components with the ability to metabolize glucose, acetate and glycine. We used this network as a starting point for further reaction elimination to generate a minimal network that was required to be viable on acetate and glycine but not on glucose. The network able to grow only on acetate and glycine had two fewer reactions than the network able to grow on all three carbon sources. One of these is a glycolytic reaction (atp + fructose-6-phosphate → adp + fructose 1,6-bisphosphate + h), the other is an anaplerotic reaction (atp + oxaloacetate → adp + co2 + phosphoenolpyruvate).

**Example 2:** We generated a minimal network that was required to synthesize *B*=63 biomass components with the ability to metabolize glucose, glutamate and pyruvate. We used this network as a starting point for further reaction elimination to generate a minimal network that was required to be viable on glutamate and pyruvate but not on glucose. The network able to grow only on glutamate and pyruvate had two fewer reactions than the network able to grow on all three carbon sources. Both of them are glycolytic reactions ((1) atp + fructose-6-phosphate → adp + fructose 1,6-bisphosphate + h, (2) glucose-6-phosphate → fructose-6-phophate).
