# Supplementary material for: Design Constraints on a Synthetic Metabolism
Source: PLoS One. 2012 Jun 29;7(6):e39903. doi: 10.1371/journal.pone.0039903 (PMC3387219; doi:10.1371/journal.pone.0039903)
Supplement: Text S3 — Examples of reactions needed to synthesize additional biomass molecules. (DOC) [file pone.0039903.s010.doc]

**Examples of reactions needed to synthesize additional biomass molecules**

These examples take a network size reduction approach to illustrate the kinds of reactions needed to synthesize additional biomass molecules.

**Example 1:** We generated a minimal network that was able to synthesize glutamate, asparagine and proline with glucose as the sole carbon source. We used this network as the starting point for further reaction elimination to generate a minimal network synthesizing glutamate and asparagine but, not proline. This resulted in the elimination of the following three reactions: (1) atp + glutamate → adp + glutamate 5-phosphate, (2) glutamate 5-phosphate + h + nadph → glutamate 5-semialdehyde + nadp + inorganic phosphate, (3) glutamate 5-semialdehyde → 1-pyrroline-5-carboxylate + h + h2o. These reactions are involved in the conversion of glutamate to proline.

**Example 2:** We generated a minimal network that was able to synthesize glutamate, arginine and adenonsine-3,5-bisphosphate in glucose. We used this network as the starting point for further reaction elimination to generate a minimal network synthesizing glutamate and arginine but not adenonsine-3,5-bisphosphate. This resulted in the elimination of the following three reactions: (1) acetyl-glutamate 5-semialdehyde + h2o → acetate + glutamate 5-semialdehyde, (2) acetyl-glutamate 5-semialdehyde --> 1-pyrroline-5-carboxylate + h + h2o, (3) 1-pyrroline-5-carboxylate + h + nadph → nadp + adenosine-3,5-bisphosphate. These reactions are involved in adenosine-3,5-bisphosphate synthesis.
